# Use of monoclonal antibody therapy for nosocomial SARS-CoV-2 infection in patients at high risk for severe COVID-19: experience from a tertiary-care hospital in Germany

**DOI:** 10.1007/s15010-021-01657-y

**Published:** 2021-07-09

**Authors:** Johanna Koehler, Barbara Ritzer, Simon Weidlich, Friedemann Gebhardt, Chlodwig Kirchhoff, Jens Gempt, Christiane Querbach, Dieter Hoffmann, Bernhard Haller, Roland M. Schmid, Jochen Schneider, Christoph D. Spinner, Roman Iakoubov

**Affiliations:** 1 Department of Internal Medicine II, School of Medicine, University Hospital Rechts der Isar, Technical University of Munich, Munich, Germany; 2 School of Medicine, Institute for Medical Microbiology, Immunology and Hygiene, Technical University of Munich, Munich, Germany; 3 Department of Traumatology, School of Medicine, University Hospital Rechts der Isar, Technical University of Munich, Munich, Germany; 4 Department of Neurosurgery, School of Medicine, University Hospital Rechts der Isar, Technical University of Munich, Munich, Germany; 5 School of Medicine, Hospital Pharmacy, University Hospital Rechts der Isar, Technical University of Munich, Munich, Germany; 6 School of Medicine, Institute of Virology, Technical University of Munich, Munich, Germany; 7 Institute of Medical Statistics and Epidemiology, Technical University of Munich, Munich, Germany

**Keywords:** COVID-19, Bamlanivimab, Casirivimab, Imdevimab, Monoclonal spike antibodies, SARS-CoV2

## Abstract

Additional treatment options for coronavirus disease (COVID-19) are urgently needed, particularly for populations at high risk of severe disease. This cross-sectional, retrospective study characterized the outcomes of 43 patients with nosocomial severe acute respiratory syndrome coronavirus-2 (SARS-CoV-2) infection with and without treatment using monoclonal SARS-CoV-2 spike antibodies (bamlanivimab or casirivimab/imdevimab). Our results indicate that treatment with monoclonal antibodies results in a significant decrease in disease progression and mortality when used for asymptomatic patients with early SARS-CoV-2 infection.

**Supplementary Information:**

The online version contains supplementary material available at 10.1007/s15010-021-01657-y.

## Introduction

Coronavirus disease (COVID-19) is caused by severe acute respiratory syndrome coronavirus-2 (SARS-CoV-2). While dexamethasone treatment reduced mortality in severe and critical COVID-19, and remdesivir treatment was associated with shortening the recovery time in hospitalized patients, additional therapy approaches are urgently needed [1, 2]. The use of monoclonal SARS-CoV-2 spike antibodies (mABs), particularly bamlanivimab (LY-CoV555), has been associated with a decrease in hospitalization frequency in outpatients with COVID-19 [3]; however, a recent report showed no significant efficacy of LY-CoV555 administration in hospitalized patients [4], possibly due to the recruitment of symptomatic patients at the later stage of disease progression and the increasing prevalence of escape mutations [5, 6].

The German government directly purchased LY-CoV555 (bamlanivimab) and REGN-CoV-2 (combination of casirivimab and imdevimab) in January 2021, making the administration of mABs possible in February 2021 (initially LY-CoV555 at 700 mg per dose, followed by REGN-CoV-2 1200 mg casirivimab and 1200 mg imdevimab per dose). The administration of mABs was possible for patients at risk of severe or critical COVID-19, initially available for hospitalized patients only within a national emergency program.

Since October 2020, there has been a significant increase in the number of SARS-CoV-2 cases in German hospitals (“second wave”), including our facility, an academic tertiary-care teaching hospital. Due to the high number of patients and increased load of infected patients and personnel, nosocomial SARS-CoV-2 infections have occurred. The detected nosocomial SARS-CoV-2 infections were mostly traced back to contacts with infected patients, visiting relatives, or care suppliers.

Starting in February 2021, we initiated administering mABs as part of the aforementioned national emergency program. The administration was performed according to the national prescribing instructions of the Federal Ministry of Health and Internal Hospital, as designed by the hospital COVID-Expert Committee.

This retrospective study majorly aimed to evaluate the early administration of mABs for nosocomial SARS-CoV-2 infections in yet asymptomatic patients at high risk for a severe course of COVID-19 disease in real-life settings.

## Methods

### Patients

We retrospectively analyzed all patients admitted to our hospital (an 1161-bedded acute and tertiary-care teaching hospital) in Munich, Germany, from September 28th, 2020 (week 40) until April 11th, 2021 (week 14) to identify nosocomial SARS-CoV-2 infections. We excluded all patients with negative SARS-CoV-2 and those admitted to our hospital for COVID-19 treatment. Additionally, we excluded all those with previously reported SARS-CoV-2 infection, positive SARS-CoV-2 serology testing (Yhlo Biosciences, Shenzhen, China; IgG or IgM), COVID-19 typical lesions on computed tomography scan, or COVID-19 typical symptoms at the time of diagnosis. Forty-three patients at risk of severe COVID-19, who were SARS-CoV-2 negative (as verified using reverse transcription-polymerase chain reaction [RT-PCR]) at hospital admission (a general SARS-CoV-2 screening on admission day since September 2020) and developed SARS-CoV-2 infection during the hospital stay.

As the study was retrospective, written informed consent was waived according to the applicable local law. Local ethics committee counseling was conducted.

### Administration of mABs

Since February 2021, we started to administer LY-CoV555 (eight cases) and REGN-CoV-2 (three cases). All patients signed the informed consent form for emergency use of mABs, as provided and required by the German government. Following a positive PCR test and a proof of negative SARS-CoV-2 serology, the available mABs were administered. LY-CoV555 (700 mg) was dissolved in 200 mL normal saline and administered as a single intravenous infusion for approximately 1 h. REGN-CoV-2 (2400 mg) was dissolved in 250 mL normal saline and administered as a single intravenous infusion for approximately 1 h.

### Statistical analysis

Descriptive analysis was performed using GraphPad Prism version 9 (GraphPad, San Diego, CA, USA). The parameters were tested for normal distribution using D’Agostino-Pearson normality test. The values were expressed as mean ± standard deviation, and statistical significance was analyzed using unpaired *t* test, when appropriate. The non-normal values were reported as median and interquartile range (IQR). Survival analysis was performed using the Kaplan–Meier method and analyzed for significant difference in distribution of adverse events over time using the log-rank/Mantel–Cox test. The starting point was the detection of SARS-CoV-2 virus, while death or ICU admission was used as a combined end-point. An additional analysis using Kaplan–Meier plot followed by log-rank/Mantel–Cox test was performed for the combined end-point dexamethasone and/or remdesivir use. The patients were censored following the discharge or transfer from the COVID ward.

## Results

A total of 43 patients were included in this study. Thirteen patients were offered an emergency administration of mABs and two patients declined; therefore, 11 patients (25.6% of total) received mABs in addition to the standard of care. Table 1 illustrates the baseline characteristics of nosocomial SARS-CoV-2 cases. The mean age did not differ between the groups, as analyzed by unpaired *t* test (71.9 ± 13.4 vs. 71.1 ± 13.7 years, *p* = 0.86); there were slightly more female than male patients. Most patients had two or more risk factors for severe COVID-19, and nearly all patients had cardiovascular risk factors. The patients did not differ in terms of the established risk factors of severe COVID-19, for example, aged 65 years or above and/or diabetes mellitus, obesity, chronic lung disease, or immunosuppression/active cancer [7]. Admission to the hospital before infection was necessary due to urgent medical conditions, mostly requiring surgical intervention (23 out of 32 patients in the conventional treatment group [71.8%] and 9 out of 11 in the mABs treatment group [81.8%]).

**Table 1:** Baseline characteristics

	Conventional treatment (*n* = 32)	Monoclonal antibodies (*n* = 11)
Demographics and history		
Sex		
Female, *n* (%)	21 (65.6)	7 (63.6)
Male, *n* (%)	11 (34.4)	4 (36.4)
Mean age, years (SD)	71.9 (13.4)	71.1 (13.7)
Risk factors, *n* (%)		
Diabetes mellitus	11 (34.4)	5 (45.5)
Obesity	7 (21.9)	2 (18.2)
Immunosuppression/active cancer	5 (15.6)	2 (18.2)
Chronic lung disease	4 (12.5)	4 (36.4)
Home oxygen therapy	0	2 (18.2)
Cardiovascular diseases	20 (62.5)	10 (90.9)
Primary admission department, *n* (%)		
Trauma surgery	10 (31.3)	0
Neurosurgery	8 (25.0)	5 (45.5)
Orthopedics	2 (6.3)	0
Visceral surgery	3 (9.4)	3 (27.3)
Vascular surgery	0	1 (9.1)
Pneumology	0	1 (9.1)
Gastroenterology	1 (3.1)	1 (9.1)
Neurology	1 (3.1)	0
Ophthalmology	1 (3.1)	0
Psychiatry	4 (12.5)	0
Gynecology and obstetrics	2 (6.3)	0

*SD* standard deviation

After the diagnosis of SARS-CoV-2 infection, all patients were treated by the designated COVID-19 personnel. All patients received the same standard of care, as recommended by current in-hospital guidelines; in particular, only supportive therapy in the early stage, while in the late stage of the disease, dexamethasone was used, as appropriate. Two patients with SARS-CoV-2 additionally received remdesivir. The mABs infusion was well tolerated, and no immediate side effects following administration of mABs were observed.

**Fig. 1:**
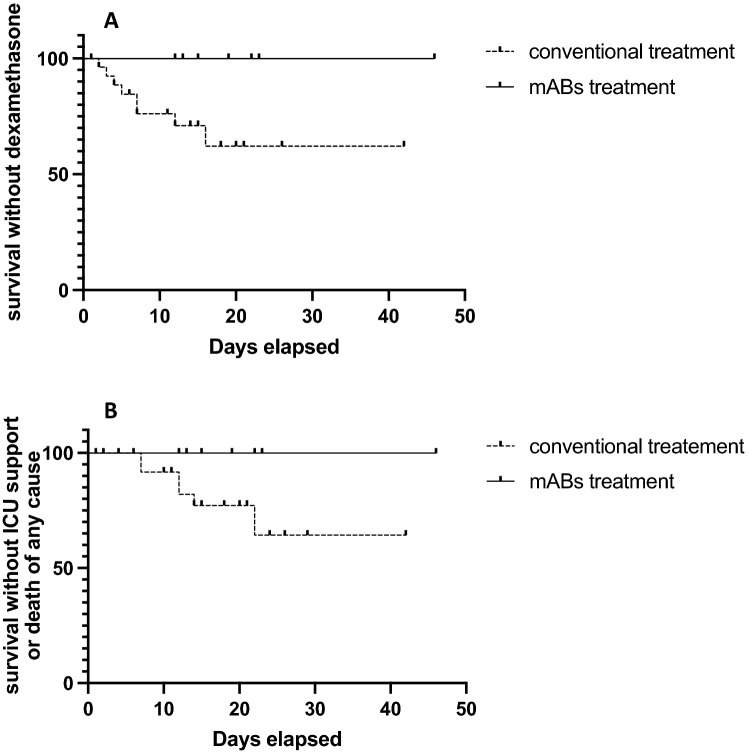
Survival analysis by Kaplan–Meier curve and log-rank (Mantel–Cox) test. **A** Significantly less patients in the mABs group require dexamethasone or remdesivir treatment due to decreased pulmonary function (8 vs. 0 cases, *p* = 0.036). **B** While indicating an improved survival of patients treated with monoclonal antibodies, the analysis does not reach significance due to small number of patients (6 vs. 0 cases, *p* = 0.068)

The total duration of the post-infection hospitalization at the COVID-19 unit did not significantly differ between the groups, as analyzed by unpaired t test (17.3 ± 3.7 vs 13.7 ± 9.7 days, *p* = 0.24), possibly due to not COVID-19-related hospitalization. Furthermore, the levels of inflammatory marker C-reactive protein (CRP) were comparable 3–5 days before virus detection and within 24 h after virus detection. Nevertheless, more adverse events occurred in the conventionally treated group as compared to the mABs treatment group (Table 2). The CRP levels in mABs treatment group were significantly lower at 7–10 days after the virus detection (60.3 ± 39.4 vs 23.7 ± 16.7 mg/L, *p* = 0.006) While only 4 out of 11 patients in the mABs treatment group developed COVID-19 typical infiltrates on computed tomographic scan (36.4%), 19 out of 32 patients in the conventional treatment group showed COVID-19-associated radiological changes (59.4%) during hospitalization. Substantially more patients in the conventional treatment group required dexamethasone or remdesivir treatment as a sign of decreased pulmonary function (8 vs. 0 patients in the mABs group), showing significant difference in distribution of event time (*p* = 0.036, log-rank/Mantel–Cox test) (Fig. 1a). Ultimately, six patients from the conventional treatment group were admitted to the intensive care unit or died during the disease course. None of the patients treated with mABs were admitted to the intensive care unit or died from any causes (Fig. 1b). Further information on the estimation of the event time distribution is available in the supplemental tables S1 and S2.

**Table 2:** Clinical and therapeutical features and outcomes of SARS-CoV-2 patients with and without mABs treatment

	Conventional treatment (*n* = 32)	Monoclonal antibodies (*n* = 11)
Total stay in the hospital post-infection days, mean ± SD	13.4 ± 9.6	17.3 ± 3.7
Radiological findings in CT-scan, *n* (%)		
Not performed	7 (21.9)	1 (9.1)
No COVID-19-typical infiltrates	6 (18.6)	6 (54.6)
Interstitial infiltrates	19 (59.4)	4 (36.4)
Pleural effusion	12 (37.5)	4 (36.4)
CRP in mg/L, median [IQR] (% of total)		
3–5 days before virus detection	104 [179] (59.4)	123 [360] (81.8)
Within 24 h after virus detection	260 [612] (84.4)	170 [567] (100)
7–10 days after virus detection	64.5 [138] (68.8)	17 [30] (100)
Therapeutic features, *n* (%)		
Demand for medical oxygen	19 (59.4)	2 (18.2)
Dexamethasone	8 (25.0)	0
Remdesivir	2 (6.3)	0
Complications, *n* (%)		
Admission to intensive care unit	5 (15.6)	0
Death of any course	6 (18.6)	0
SARS-CoV-2, variants of concern, *n* (%)		
B.1.1.7 (alpha)	1 (3)	2 (18)
B.1.177	1(3)	0
B.1.258.17	1 (3)	2 (18)

*SARS-CoV-2* severe acute respiratory syndrome coronavirus-2, *mABs* monoclonal SARS-CoV-2 spike antibodies, *SD* standard deviation, *CT* computed tomography, *COVID-19* coronavirus disease, *CRP* C-reactive protein

We also analyzed the samples for variants of concern (VOC), a particular concern in mABs treated patients. We identified three VOC alpha B.1.1.7, and the other sequenced strains were classified as three B.1.258.17 and one B1.177 (Table 2).

## Discussion

Our study found that treatment using mABs in nosocomial SARS-CoV-2 infection seems to be a safe and effective option for patients at risk for severe COVID-19. While the time spent in hospital was not significantly reduced (partly due to frequently changing quarantine requirements for SARS-CoV-2 in Germany), the lower number of radiologically detected COVID-19 infections and the absence of a severe course of COVID-19 disease were quite remarkable. Because of the in-house transmission of SARS-CoV-2 infection, we were able to administer the monoclonal antibodies shortly after an infection, in all cases before the development of any symptoms. While we only treated hospitalized patients, our data differ substantially from those of previously published studies on monoclonal antibodies in hospitalized patients. In contrast to the publication by ACTIV-3/TICO LY-CoV555 Study Group [4], which showed no clinical benefit of mABs administration in patients with already present symptoms of COVID-19, we only included asymptomatic nosocomial SARS-CoV-2-infected cases directly after detection of SARS-CoV-2 infection. Our early intervention is rather comparable to the recently published outpatient studies [3], indicating that in an outpatient setting, fewer patients required hospitalization following administration of monoclonal antibodies (1 out of 101 [1%] vs. 9 out of 143 [6.3%] for LY-CoV555 700 mg/dose); this effect was even more prominent in patients over 65 years of age and with a body mass index of 35 or more (4 out of 95 [4%] vs. 7 out of 48 [15%], for all doses of LY-CoV555). Comparable results were also shown in a retrospective case–control study, again in an outpatient setting [8].

Our study had several limitations. First, the sample size was small, and the analysis was based on retrospectively collected data; therefore, only univariate analysis without adjustment for potential confounders was possible. Second, due to the restricted availability of monoclonal antibodies, as only available within a German national emergency program, two different antibody treatments were administered, limiting the comparability of the results. Third, the administration of mABs occurred mostly in February and March 2021, while conventionally treated patients were mostly hospitalized during the fall season in our hospital, causing a possible shift in treatment options. Furthermore, starting in 2021, new virus variants (variants of concern), particularly B 1.1.7, rapidly increased in Germany, making the interpretation of the quite promising data more difficult.

Nevertheless, considering the scarce resources of intensive care during the COVID-19 pandemic, treatment with monoclonal antibodies might be considered an option in the early course of COVID-19 disease, particularly in unvaccinated patients at risk for severe COVID-19. Further studies investigating the optimal criteria for the administration of monoclonal antibodies are necessary to improve the outcome for patients with COVID-19.

## Supplementary Information

Below is the link to the electronic supplementary material.

Supplementary file1 (DOCX 21 KB)
