# Supplementary material for: Use of monoclonal antibody therapy for nosocomial SARS-CoV-2 infection in patients at high risk for severe COVID-19: experience from a tertiary-care hospital in Germany
Source: Infection. 2021 Jul 9;49(6):1313–8. doi: 10.1007/s15010-021-01657-y (PMC8269399; doi:10.1007/s15010-021-01657-y)
Supplement: Supplementary file 1 — Supplementary file1 (DOCX 21 KB) [file 15010_2021_1657_MOESM1_ESM.docx]

**Supplemental Tables**

**Table S1:** Calculation for the Kaplan-Meier estimate of the survival function. Calculation for the end point ICU or death of any cause.

|  |  |  |  | Cumulative Proportion Surviving at the Time | |  |  |
| --- | --- | --- | --- | --- | --- | --- | --- |
| Group | Patient No. | Days | Status | Estimate | Std. Error | No. of Cumulative Events | No. of Remaining Cases |
| mABs | 1 | 12 | 0 |  |  | 0 | 11 |
|  | 2 | 13 | 0 |  |  | 0 | 10 |
|  | 3 | 15 | 0 |  |  | 0 | 9 |
|  | 4 | 15 | 0 |  |  | 0 | 8 |
|  | 5 | 15 | 0 |  |  | 0 | 7 |
|  | 6 | 19 | 0 |  |  | 0 | 6 |
|  | 7 | 19 | 0 |  |  | 0 | 5 |
|  | 8 | 22 | 0 |  |  | 0 | 4 |
|  | 9 | 22 | 0 |  |  | 0 | 3 |
|  | 10 | 23 | 0 |  |  | 0 | 2 |
|  | 11 | 46 | 0 |  |  | 0 | 1 |
| Conventional treatment | 1 | 1 | 0 |  |  | 0 | 31 |
|  | 2 | 1 | 0 |  |  | 0 | 30 |
|  | 3 | 1 | 0 |  |  | 0 | 29 |
|  | 4 | 1 | 0 |  |  | 0 | 28 |
|  | 5 | 1 | 0 |  |  | 0 | 27 |
|  | 6 | 2 | 0 |  |  | 0 | 26 |
|  | 7 | 4 | 0 |  |  | 0 | 25 |
|  | 8 | 6 | 0 |  |  | 0 | 24 |
|  | 9 | 7 | 1 |  |  | 1 | 23 |
|  | 10 | 7 | 1 | 0.917 | 0.056 | 2 | 22 |
|  | 11 | 10 | 0 |  |  | 2 | 21 |
|  | 12 | 11 | 0 |  |  | 2 | 20 |
|  | 13 | 11 | 0 |  |  | 2 | 19 |
|  | 14 | 12 | 1 |  |  | 3 | 18 |
|  | 15 | 12 | 1 | 0.820 | 0.082 | 3 | 17 |
|  | 16 | 14 | 1 | 0.772 | 0.090 | 4 | 16 |
|  | 17 | 14 | 0 |  |  | 4 | 15 |
|  | 18 | 15 | 0 |  |  | 4 | 14 |
|  | 19 | 15 | 0 |  |  | 4 | 13 |
|  | 20 | 15 | 0 |  |  | 4 | 12 |
|  | 21 | 15 | 0 |  |  | 4 | 11 |
|  | 22 | 18 | 0 |  |  | 4 | 10 |
|  | 23 | 18 | 0 |  |  | 4 | 9 |
|  | 24 | 20 | 0 |  |  | 4 | 8 |
|  | 25 | 20 | 0 |  |  | 4 | 7 |
|  | 26 | 21 | 0 |  |  | 4 | 6 |
|  | 27 | 22 | 1 | 0.643 | 0.139 | 5 | 5 |
|  | 28 | 24 | 0 |  |  | 5 | 4 |
|  | 29 | 24 | 0 |  |  | 5 | 3 |
|  | 30 | 26 | 0 |  |  | 5 | 2 |
|  | 31 | 29 | 0 |  |  | 5 | 1 |
|  | 32 | 42 | 0 |  |  | 5 | 0 |

**Table S2:** Calculation for the Kaplan-Meier estimate of the survival function. Calculation for the end point use of dexamethasone or remdesivir.

|  |  |  |  | Cumulative Proportion Surviving at the Time | |  |  |
| --- | --- | --- | --- | --- | --- | --- | --- |
| Group | Patient No. | Days | Status | Estimate | Std. Error | No. of Cumulative Events | No. of Remaining Cases |
| mABs | 1 | 12 | 0 |  |  | 0 | 11 |
|  | 2 | 13 | 0 |  |  | 0 | 10 |
|  | 3 | 15 | 0 |  |  | 0 | 9 |
|  | 4 | 15 | 0 |  |  | 0 | 8 |
|  | 5 | 15 | 0 |  |  | 0 | 7 |
|  | 6 | 19 | 0 |  |  | 0 | 6 |
|  | 7 | 19 | 0 |  |  | 0 | 5 |
|  | 8 | 22 | 0 |  |  | 0 | 4 |
|  | 9 | 22 | 0 |  |  | 0 | 3 |
|  | 10 | 23 | 0 |  |  | 0 | 2 |
|  | 11 | 46 | 0 |  |  | 0 | 1 |
| Conventional treatment | 1 | 1 | 0 |  |  | 0 | 31 |
|  | 2 | 1 | 0 |  |  | 0 | 30 |
|  | 3 | 1 | 0 |  |  | 0 | 29 |
|  | 4 | 1 | 0 |  |  | 0 | 28 |
|  | 5 | 1 | 0 |  |  | 0 | 27 |
|  | 6 | 2 | 1 | 0.963 | 0.036 | 1 | 26 |
|  | 7 | 2 | 0 |  |  | 1 | 25 |
|  | 8 | 3 | 1 | 0.924 | 0.051 | 2 | 24 |
|  | 9 | 4 | 1 | 0.886 | 0.062 | 3 | 23 |
|  | 10 | 4 | 0 |  |  | 3 | 22 |
|  | 11 | 5 | 1 | 0.846 | 0.071 | 4 | 21 |
|  | 12 | 6 | 0 |  |  | 4 | 20 |
|  | 13 | 7 | 1 |  |  | 5 | 19 |
|  | 14 | 7 | 1 | 0.761 | 0.086 | 6 | 18 |
|  | 15 | 7 | 0 |  |  | 6 | 17 |
|  | 16 | 7 | 0 |  |  | 6 | 16 |
|  | 17 | 11 | 0 |  |  | 6 | 15 |
|  | 18 | 12 | 1 | 0.710 | 0.094 | 7 | 14 |
|  | 19 | 12 | 0 |  |  | 7 | 13 |
|  | 20 | 14 | 0 |  |  | 7 | 12 |
|  | 21 | 14 | 0 |  |  | 7 | 11 |
|  | 22 | 15 | 0 |  |  | 7 | 10 |
|  | 23 | 15 | 0 |  |  | 7 | 9 |
|  | 24 | 15 | 0 |  |  | 7 | 8 |
|  | 25 | 16 | 1 | 0.622 | 0.117 | 8 | 7 |
|  | 26 | 18 | 0 |  |  | 8 | 6 |
|  | 27 | 18 | 0 |  |  | 8 | 5 |
|  | 28 | 20 | 0 |  |  | 8 | 4 |
|  | 29 | 20 | 0 |  |  | 8 | 3 |
|  | 30 | 21 | 0 |  |  | 8 | 2 |
|  | 31 | 26 | 0 |  |  | 8 | 1 |
|  | 32 | 42 | 0 |  |  | 8 | 0 |
